# Neuropeptide S-Mediated Modulation of Prepulse Inhibition Depends on Age, Gender, Stimulus-Timing, and Attention

**DOI:** 10.3390/ph14050489

**Published:** 2021-05-20

**Authors:** Wei Si, Xiaobin Liu, Hans-Christian Pape, Rainer K. Reinscheid

**Affiliations:** 1 Department of Pharmaceutical Sciences, University of California Irvine, Irvine, CA 92697, USA; wsiuci@gmail.com (W.S.); Xiaobin.Liu@unthsc.edu (X.L.); 2 Institute of Physiology I, Westfälische-Wilhelms University, 48149 Münster, Germany; Hans-Christian.Pape@ukmuenster.de; 3 Institute of Pharmacology and Toxicology, Friedrich-Schiller University, 07747 Jena, Germany

**Keywords:** NPSR1, NPS, knockout mouse, PPI, schizophrenia, clozapine, MK-801

## Abstract

Conflicting reports about the role of neuropeptide S (NPS) in animal models of psychotic-like behavior and inconsistent results from human genetic studies seeking potential associations with schizophrenia prompted us to reevaluate the effects of NPS in the prepulse inhibition (PPI) paradigm in mice. Careful examination of NPS receptor (NPSR1) knockout mice at different ages revealed that PPI deficits are only expressed in young male knockout animals (<12 weeks of age), that can be replicated in NPS precursor knockout mice and appear strain-independent, but are absent in female mice. PPI deficits can be aggravated by MK-801 and alleviated by clozapine. Importantly, treatment of wildtype mice with a centrally-active NPSR1 antagonist was able to mimic PPI deficits. PPI impairment in young male NPSR1 and NPS knockout mice may be caused by attentional deficits that are enhanced by increasing interstimulus intervals. Our data reveal a substantial NPS-dependent developmental influence on PPI performance and confirm a significant role of attentional processes for sensory-motor gating. Through its influence on attention and arousal, NPS appears to positively modulate PPI in young animals, whereas compensatory mechanisms may alleviate NPS-dependent deficits in older mice.

## 1. Introduction

The neuropeptide S (NPS) system has received considerable attention as either a genetic risk factor or potential therapeutic target for several psychiatric disorders, including anxiety and schizophrenia. Behavioral and physiological effects of NPS have been investigated in several preclinical animal models of psychosis-related behaviors, and two genetic association studies have investigated a possible link between NPS receptor (NPSR1) variants with schizophrenia in humans.

Due to NPS-induced arousal-promoting effects, it was initially investigated for potential therapeutic effects in the most commonly used animal model in schizophrenia research, i.e., prepulse inhibition (PPI). Our group first reported ameliorating effects of central NPS administration on MK-801-induced pathological, neurochemical, and behavioral disruptions. Importantly, NPS was shown to reverse MK-801-induced disruption of PPI and displayed a pharmacological profile similar to atypical antipsychotics [1]. Reversal of MK-801-induced cognitive deficits by central NPS injection was demonstrated independently [2,3]. Surprisingly, male NPSR1 knockout (KO) mice appeared to show no phenotypical changes in PPI performance, although PPI levels were consistently—but insignificantly—lower in NPSR1 knockout (KO) mice than in wildtype (WT) littermates [4]. Zhu et al. [5] generated an independent NPSR1 KO line and reported significantly reduced acoustic startle responses in male KO mice without apparent effects on PPI, although a standard PPI profile was not applied in the study. These observations were confirmed by Fendt et al. [6], who reported reduced acoustic startle responses and no obvious differences between NPSR1 KO and WT mice using a standard PPI paradigm with 4 different prepulse levels. In the same study [6], NPSR1 KO mice also displayed a non-significant trend to lower PPI levels, similar to our own observations [4]. In both studies, mice were derived from the original NPSR1 KO line [7], albeit on different genetic backgrounds (Table 1). Female NPSR1 KO mice displayed no phenotypical changes in PPI or acoustic startle [5]. The studies by Duangdao et al. and Fendt et al. tested mice of a wider age range (8–15 weeks [4]; 12–25 weeks [6], respectively), whereas Zhu et al. used 8–10 week old animals [5]. Parameters and results pertaining to NPS-dependent acoustic startle and PPI are summarized in Table 1.

In summary, although pharmacological studies suggested positive effects of NPS on PPI performance, data from KO mice did either not confirm a functional role of NPS in sensory-motor gating or yielded inconclusive results.

Genetic variants of NPSR1 were also investigated for a link with schizophrenia. Two candidate gene studies focused on a non-synonymous single-nucleotide polymorphism (SNP rs324981, T/A) that has been associated with panic disorder, fear processing, anxiety disorders, impulsivity, and stress responsiveness [8,9,10,11,12,13,14,15,16]. The polymorphism changes the amino acid sequence of the receptor at position 107 (Ile to Asn), with a 5–10-fold higher agonist efficacy in the NPSR1-Ile^107^ variant compared to NPSR1-Asn^107^ [17]. Our own investigation detected no association of NPSR1 rs324981 alleles with schizophrenia in a Japanese case/control cohort [8]. However, Lennertz et al. [18] reported significant association of the low-functioning NPSR1-Asn^107^ variant with schizophrenia in a German case/control cohort. In functional tests, attenuated verbal memory consolidation in homozygous NPSR1-Asn^107^ carriers was detected while memory acquisition was not influenced by NPSR1 genotypes. Ile^107^ carriers in the schizophrenia patient cohort displayed significantly reduced startle amplitudes without changes in PPI or startle habituation. We had previously reported that central NPS administration in mice promotes memory consolidation, independent of affective stimulus modality [19]. The study in schizophrenia patients, therefore, corroborated earlier observations from rodent models, suggesting NPS-dependent modulation of memory consolidation. However, the role of NPS signaling in PPI remains less clear.

Disruption of PPI has been considered an endophenotype of schizophrenia in humans (but also other psychiatric disorders; for review see [20]) and the most reliable operational paradigm for the measurement of sensorimotor gating mechanisms, that is widely used as a translational model in experimental animals to screen for novel antipsychotic drugs. PPI deficits in schizophrenia patients are thought to reflect dysfunctions of sensorimotor gating, probably caused by sensory flooding and cognitive fragmentation [21,22]. Furthermore, patients diagnosed with schizophrenia exhibit deficient attentional processing and cognitive functions [23]. It has been suggested that PPI occurs involuntarily but can be modulated by controlled attentional processes. In humans, PPI at short interstimulus intervals (ISI) occurs by largely automatic processing, while PPI at longer ISI (>120 ms) involves both attentional and automatic processing [24,25,26]. With impaired attentional control, schizophrenia patients fail to present any attentional modulation of PPI and studies suggest that attention deficits may contribute to disrupted PPI in schizophrenia [23,27].

To clarify the role of endogenous NPS signaling in sensorimotor gating, we launched a systematic investigation in mice, controlling for age, sex, genetic background, and stimulus-timing, by using prepulse-inhibition as a prototypical behavioral assay. Genetic mouse models were complemented by pharmacological tools in order to investigate effects of disrupted NPS signaling on PPI performance. In addition, we examined NPS-related effects on PPI-disrupting drugs and conducted phenotype-rescue experiments with the atypical antipsychotic clozapine in NPSR1 KO mice. Our results indicate that age, gender, and stimulus-timing have significant influences on PPI performance in mice with genetically or pharmacologically disrupted NPS signaling. 

## 2. Results

### 2.1. NPS-Dependent Age Effect on PPI

**Figure 1:**
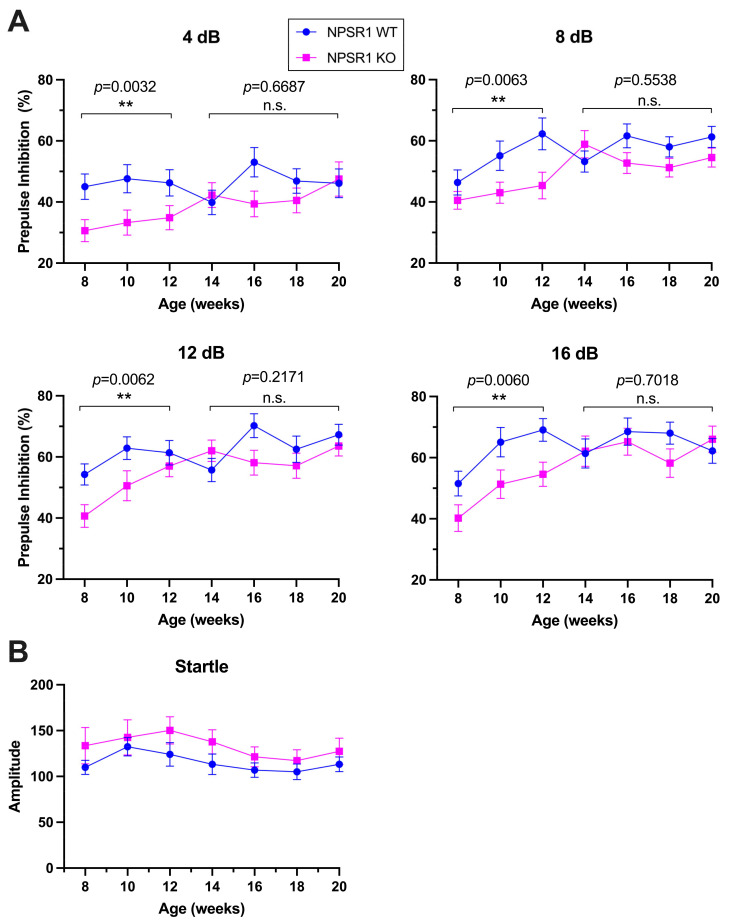
Dissociation of age effects on (**A**) PPI and (**B**) startle reflex in male C57Bl/6J-NPSR1 WT and KO mice. (**A**) One-way ANOVA showed no age effect on PPI in WT mice, whereas KO mice displayed significant age effects between 8 and 20 weeks at prepulse intensities of 4, 8, 12, and 16 dB. Two-way ANOVA revealed that PPI in KO mice was significantly lower than in WT controls between 8 and 12 weeks of age at all prepulse intensities, whereas no difference between genotypes was detected between 14 and 20 weeks of age. ** *p* < 0.01; n.s., not significant. (**B**) No age effect on startle reflex was detected in WT and KO mice and between genotypes. WT, *n* = 11; KO, *n* = 9.

To study a potential effect of age on PPI in mice ranging from early adolescence to adulthood, we used male C57Bl/6J-NPSR1 WT and KO mice between 8 and 20 weeks of age. As shown in Figure 1, repeated-measures ANOVA revealed no age effect on PPI at all prepulse intensities in C57Bl/6J-NPSR1 WT mice, whereas C57Bl/6J-NPSR1 KO mice displayed significant age effects across late adolescence and young adulthood at prepulse intensities of 8, 12, and 16 dB (F_8dB_(6, 63) = 2.563, *p* = 0.0277; F_12dB_(6, 63) = 2.760, *p* = 0.0191; F_16dB_(6, 63) = 3.142, *p* = 0.0093). Moreover, two-way ANOVA indicated significantly lower PPI in KO mice compared to WT controls between 8 and 12 weeks of age at all prepulse intensities (Figure 1) (F_4dB_(1, 50) = 9.58, *p* = 0.0032; F_8dB_(1, 52) = 8.10, *p* = 0.0063; F_12dB_(1, 51) = 8.15, *p* = 0.0062; F_16dB_(1, 52) = 8.21, *p* = 0.0060), and no difference between genotypes at 14–20 weeks of age (F_4dB_(1, 68) = 0.18, *p* = 0.6687; F_8dB_(1, 67) = 0.35, *p* = 0.5583; F_12dB_(1, 68) = 1.55, *p* = 0.2171; F_16dB_(1, 68) = 0.15, *p* = 0.7018) without interaction of age × genotype.

No age effect on acoustic startle reflex was detected in both male C57Bl/6J-NPSR1 WT (F(6, 56) = 1.581, *p* = 0.1696) and KO mice (F(6, 63) = 0.8078, *p* = 0.5677) between 8 and 20 weeks of age (Figure 1B). Two-way ANOVA showed no effect of genotype (F_genotype_(1,19) = 2.1, *p* = 0.1583) or interaction of age x genotype.

Taken together, significant age-dependent changes in PPI across the investigated developmental period (8–20 weeks) were only observed in NPSR1 KO, and significant effects of genotype were most apparent in adolescent and young adult mice (8–12 weeks). Hence, all following experiments were performed with 8–12 week old mice, regardless of genetic background or gender. 

### 2.2. Comparison of PPI in NPSR1 and NPS KO Mice

**Figure 2:**
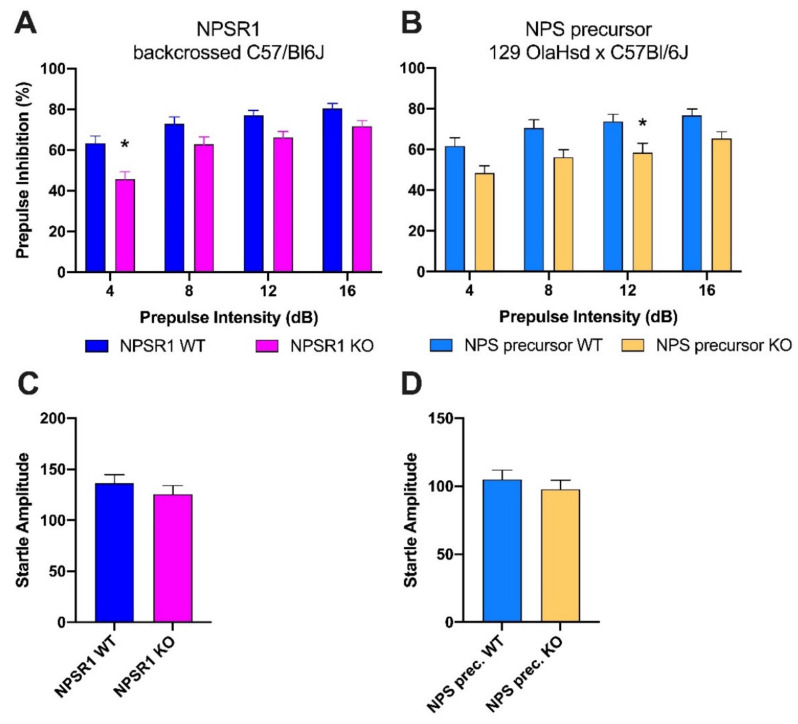
PPI in (**A**) backcrossed male C57Bl/6J-NPSR1 WT and KO mice, and (**B**) male NPS precursor WT and KO mice. Two-way ANOVA showed significant differences in PPI between C57Bl/6J-NPSR1 WT and KO mice, and NPS precursor WT and KO mice, respectively; * *p* < 0.05 vs. WT controls; Bonferroni’s post hoc test after significant two-way ANOVA. WT, *n* = 10; KO, *n* = 10. (**C**,**D**) Startle reflex. No difference in startle reflex between (**C**) backcrossed C57Bl/6J-NPSR1 WT and KO mice, or between (**D**) NPS precursor WT and KO mice. WT, *n* = 10; KO, *n* = 10.

Two further control experiments were carried out to confirm that absence of NPS signaling, and no particular strain- or genetic-model-dependent phenotypes are responsible for the observed PPI deficits in adolescent NPSR1 KO mice. In the first set of experiments, we used a separate strain of backcrossed C57Bl/6J-NPSR1 WT and KO mice that had been generated in house. The second control experiment compared PPI performance in NPS precursor WT and KO mice on a hybrid 129OlaHsd x C57Bl/6J background (Figure 2A,B). Two-way ANOVA indicated significant main effects of genotype and prepulse intensity for backcrossed C57Bl/6J-NPSR1 mice (F_genotype_(1, 72) = 16.16, *p* = 0.0001; F_prepulse intensity_(3, 72) = 10.44, *p* < 0.0001) and NPS precursor WT and KO mice (F_genotype_(1, 72) = 20.45, *p* < 0.0001; F_prepulse intensity_(3, 72) = 5.12, *p* = 0.0029), suggesting that absence of either the peptide ligand or the receptor protein results in PPI disruption in adolescent male mice (age range 8–10 weeks). Female NPS precursor WT and KO mice displayed no difference in PPI (data not shown).

As shown in Figure 2C,D, backcrossed C57Bl/6J-NPSR1 KO mice (Student’s *t*-test: t(18) = 0.6393, *p* = 0.5307) and NPS precursor KO mice (Student’s *t*-test: *t*(18) = 0.4886, *p* = 0.6310) both showed similar baseline startle reflex amplitudes compared to their respective WT controls.

### 2.3. Gender-Specific Effects and PPI Disruption by MK-801

**Figure 3:**
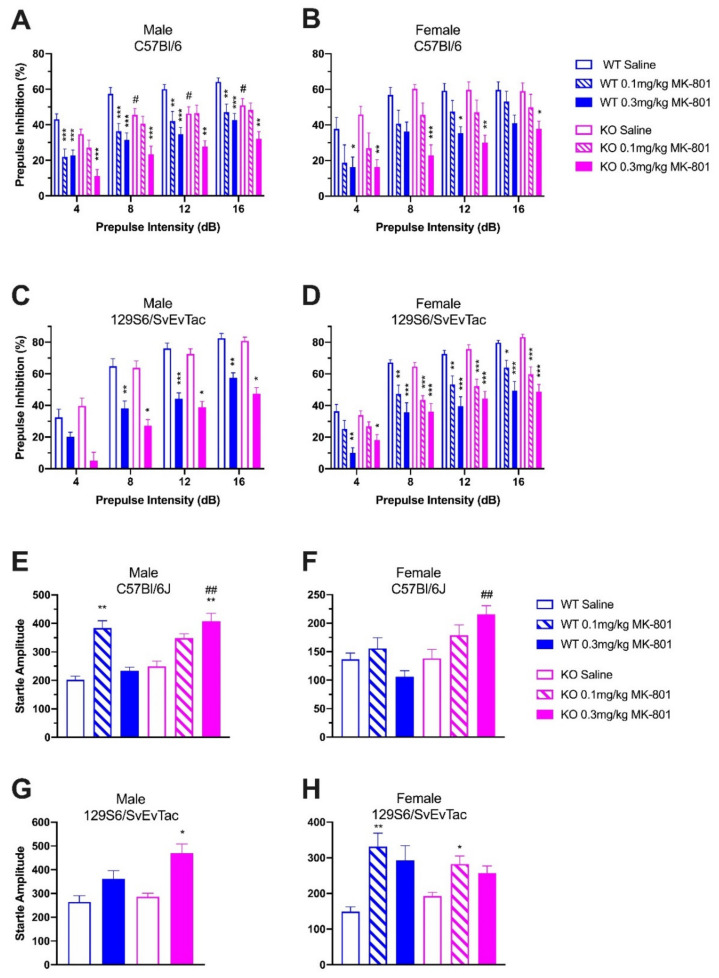
Influence of strain and gender on PPI and startle responses. MK-801-induced PPI disruption in (**A**) male and (**B**) female C57Bl/6J-NPSR1 WT and KO mice, and (**C**) male and (**D**) female 129S6/SvEvTac-NPSR1 WT and KO mice. Male, but not female, NPSR1 KO mice treated with saline or 0.3 mg/kg MK-801 showed attenuated PPI compared to WT controls with the same treatment in both strains; * *p* < 0.05, ** *p* < 0.01, *** *p* < 0.001 vs. saline-treated group within the same genotype; # *p* < 0.05 vs. WT saline-treated group; Bonferroni’s post hoc test after significant two-way ANOVA. Startle reflex in (**E**) male and (**F**) female C57Bl/6J-NPSR1 WT and KO mice, and (**G**) male and (**H**) female 129S6/SvEvTac-NPSR1 WT and KO mice after treatment with saline or MK-801. Male and female mice of both strains showed an increase in startle amplitude in response to MK-801, independent of genetic background. No difference in baseline startle amplitude between WT and KO mice within the same strain was detected; * *p* < 0.05, ** *p* < 0.01 vs. saline-treated group within the same genotype; Dunnett’s post hoc tests after significant one-way ANOVA. ## *p* < 0.01 vs. WT 0.3 mg/kg MK-801-treated group; *t* test). Animal numbers: (A and E) WT, *n* = 12–15; KO, *n* = 13–14; (B and F) WT, *n* = 10; KO, *n* = 10; (C and G) WT, *n* = 9; KO, *n* = 11–16; (D and H) WT, *n* = 8–12; KO, *n* = 12–13.

To study the effect of absent NPS signaling on PPI and MK-801-induced PPI disruption, and to investigate potential effects of gender and genetic background, male and female C57Bl/6J- and 129S6/SvEvTac-NPSR1 WT and KO mice were treated with saline or MK-801 (0.1, 0.3 mg/kg) before PPI tests (Figure 3). In male C57Bl/6J mice, two-way ANOVA (prepulse intensity × treatment) indicated strong inhibitory effects of MK-801 on PPI in both WT (F(2, 144) = 47.60, *p* < 0.0001) and KO mice (F(2, 148) = 30.87, *p* < 0.0001; Figure 3A). In WT mice, post hoc tests showed significant differences between saline- and 0.1 mg/kg MK-801-treated groups (*p* < 0.001 at 4 and 8 dB, *p* < 0.01 at 12 and 16 dB), as well as saline- and 0.3 mg/kg MK-801-treated groups (*p* < 0.001 at all prepulse levels). In KO mice, however, significant differences were only found between saline- and 0.3 mg/kg MK-801-treated animals (*p* < 0.001 at 4 and 8 dB, *p* < 0.01 at 12 and 16 dB).

Saline-treated male C57Bl/6J-NPSR1 KO mice showed consistently lower PPI than WT controls. Two-way ANOVA (prepulse intensity × genotype) indicated significant differences between the genotypes (F(1, 104) = 27.28, *p* < 0.0001), and Bonferroni post hoc tests indicated significant differences between WT and KO mice at 8, 12, and 16 dB prepulse intensities (*p* < 0.05). Treatment with 0.1 mg/kg MK-801 suppressed PPI with no remaining difference between WT and KO mice (F(1, 96) = 1.41, *p* = 0.2376). After treatment with a higher dose of MK-801 (0.3 mg/kg), PPI in KO mice was more strongly disrupted than in WT controls (F(1, 92) = 11.66, *p* = 0.001).

In female C57Bl/6J mice, treatment with MK-801 also strongly suppressed PPI in both WT (F(2, 108) = 13.08, *p* < 0.0001) and KO (F(2, 108) = 27.64, *p* < 0.0001) mice (Figure 3B). However, there were no significant differences between genotypes when comparing the same pharmacological treatment groups. Female saline-treated WT mice showed similar PPI as saline-treated KO mice (F(1, 72) = 0.75, *p* = 0.3884). No significant differences were found between WT and KO groups after treatment with 0.1 mg/kg MK-801 (F(1, 72) = 0.21, *p* = 0.6483) or 0.3 mg/kg MK-801 (F(1, 72) = 2.60, *p* = 0.1113), respectively.

In male 129S6/SvEvTac mice, MK-801 also produced strong disruption of PPI in both WT (F(1,52) = 58.19, *p* < 0.0001) and NPSR1 KO (F(1,56) = 56.96, *p* < 0.0001) groups (Figure 3C). Similar to male C57Bl/6J mice, saline-treated male 129S6/SvEvTac-NPSR1 KO mice showed consistently lower PPI than saline-treated 129S6/SvEvTac-NPSR1 WT mice (F(1,40)] = 3.95, *p* = 0.0537). Similarly, treatment with MK-801 (0.3 mg/kg) produced significantly stronger disruption of PPI in 129S6/SvEvTac-NPSR1 KO mice than in WT controls (F(1,40) = 7.58, *p* = 0.0089).

In female 129S6/SvEvTac mice, treatment with MK-801 also strongly inhibited PPI in both WT (F(2, 108) = 39.53, *p* < 0.0001) and KO (F(2, 136) = 58.03, *p* < 0.0001) mice (Figure 3D). However, similar to female C57Bl/6J-NPSR1 WT and KO mice, there were no significant differences between the genotypes that received the same treatment. Two-way ANOVA indicated similar PPI between female 129S6/SvEvTac-NPSR1 WT and KO groups after saline (F(1, 88) = 0.04, *p* = 0.8434), 0.1 mg/kg MK-801 (F(1, 92) = 0.33, *p* = 0.5662), or 0.3 mg/kg MK-801 (F(1, 64) = 0.75, *p* = 0.3903) treatment.

In addition, startle amplitudes were compared between NPSR1 WT and KO mice across the same treatment groups in C57Bl/6J and 129S6/SvEvTac mice, respectively. No difference in startle amplitude was detected between saline-treated WT and KO mice in both strains and genders (Figure 3E–H). Male (Student’s *t* test: *t*(22) = 2.981, *p* = 0.0069) and female (Student’s *t* test: *t*(18) = 3.625, *p* = 0.0019) C57Bl/6J-NPSR1 KO mice treated with 0.3 mg/kg MK-801 showed significantly higher startle amplitude than WT controls treated with the same dose of MK-801, suggesting an increased sensitivity of NPSR1 KO mice to the high dose of MK-801 (0.3 mg/kg).

Finally, acoustic startle reflexes were compared between saline- and MK-801-treated mice within the same genotype. One-way ANOVA and Dunnett’s post hoc tests indicated that MK-801 enhanced startle amplitude to a varying extent in NPSR1 WT and KO mice of both genders and strains. In male C57Bl/6J mice, one-way ANOVA showed significant effects of MK-801 on startle reflex in both NPSR1 WT (F(2, 35) = 10.37, *p* = 0.0003) and KO mice (F(2, 37) = 5.495, *p* = 0.0069). However, MK-801 produced little effect on startle reflex in female C57Bl/6J-NPSR1 WT (F(2, 27) = 1.752, *p* = 0.1926) and KO mice (F(2, 27) = 1.939, *p* = 0.1634). In male 129S6/SvEvTac mice, 0.3 mg/kg MK-801-treated KO animals showed significantly higher startle responses compared to saline-treated KO mice (Student’s *t* test: *t*(10) = 2.340, *p* = 0.0414). Similarly, in female 129S6/SvEvTac mice, MK-801 significantly increased startle response in both the NPSR1 WT (F(2, 27) = 5.926, *p* = 0.0074) and KO groups (F(2, 34) = 4.720, *p* = 0.0091).

Independent of genetic background, absence of NPS signaling appears to attenuate PPI and increase vulnerability of PPI disruption by NMDA-receptor antagonists only in male mice. These data, therefore, support a gender-specific role of NPS in sensorimotor processing.

### 2.4. Effect of SHA 68 on MK-801-Induced PPI Disruption in Wildtype Mice

To confirm the importance of intact NPS signaling for sensorimotor gating, we employed a pharmacological approach in wildtype mice of both strains used in this study. The NPSR1 antagonist SHA 68 was used to determine if blockade of NPSR1 signaling could aggravate MK-801-induced PPI disruption in both male C57Bl/6J and 129S6/SvEvTac WT mice. We used a moderate dose of MK-801 (0.1 mg/kg) in order to detect potential additive effects on PPI performance after MK-801 and SHA 68 co-administration.

**Figure 4:**
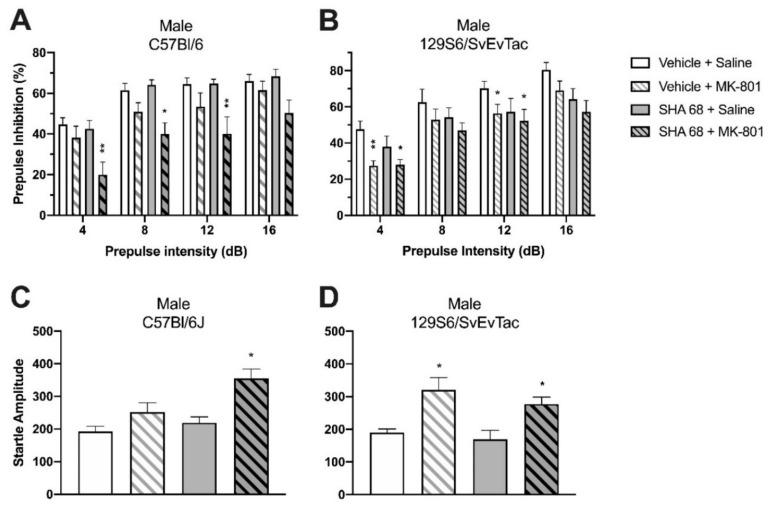
Effect of SHA 68 on 0.1 mg/kg MK-801-induced PPI disruption in male (**A**) C57Bl/6J and (**B**) 129S6/SvEvTac WT mice. (**A**) In C57Bl/6J mice, SHA 68 pretreatment aggravated MK-801-induced PPI disruption. (**B**) In 129S6/SvEvTac mice, SHA 68 alone significantly decreased PPI compared with saline-treated controls; * *p* < 0.05, ** *p* < 0.01 vs. ‘vehicle + saline’-treated group; Bonferroni’s post hoc test after significant two-way ANOVA. (**C**,**D**) Startle reflex. MK-801 alone or co-administration of MK-801 + SHA 68 significantly increased startle amplitude in male (**C**) C57Bl/6J and (**D**) 129S6/SvEvTac WT mice; * *p* < 0.05 vs. ‘vehicle + saline’-treatment group; t-test. Animal numbers: (**A**,**C**) *n* = 9–11; (**B**,**D**) *n* = 6.

In C57Bl/6J mice, PPI was significantly disrupted by administration of MK-801 (0.1 mg/kg i.p.). Two-way ANOVA (prepulse intensity × treatment) revealed significant differences between the ‘vehicle + saline’ group and ‘vehicle + MK-801′ group (F(1, 72) = 6.61, *p* = 0.0122, Figure 4A). Administration of SHA 68 alone (50 mg/kg i.p.) did not show any effect on PPI compared to ‘vehicle + saline’-treated animals (F(1,68) = 0.11, *p* = 0.7369). However, pretreatment with SHA 68 significantly aggravated MK-801-induced PPI deficits. PPI of ‘SHA 68 + MK-801′-treated animals was significantly lower than in ‘vehicle + saline’-treated mice (F(1, 76) = 31.81, *p* < 0.0001), as well as ‘vehicle + MK-801′-treated animals (F(1, 76) = 9.48, *p* = 0.0029). Bonferroni post hoc tests indicated significant differences between ‘SHA 68 + MK-801′ and ‘vehicle + saline’ groups at prepulse intensities of 4 dB (*p* < 0.01), 8 dB (*p* < 0.05), and 12 dB (*p* < 0.01).

In 129S6/SvEvTac WT mice, PPI was also disrupted by administration of MK-801. Treatment with ‘vehicle + MK-801′ showed a strong suppressing effect on PPI compared to the ‘vehicle + saline’-treated group (F(1, 68) = 28.05, *p* < 0.0001, Figure 4B). Bonferroni post hoc tests indicated significant differences between ‘vehicle + saline’ and ‘vehicle + MK-801′-treated groups at prepulse intensities of 4 dB (*p* < 0.01) and 12 dB (*p* < 0.05). Unlike C57Bl/6J mice, however, treatment with SHA 68 alone significantly decreased PPI compared to the ‘vehicle + saline’ group (F(1, 68) = 8.53, *p* = 0.0047). PPI in the ‘vehicle + MK-801′ treatment group was similar to that of ‘SHA 68 + MK-801′-treated animals (F(1, 64) = 0.81, *p* = 0.3730), suggesting little additive effect of SHA 68 on MK-801-induced PPI disruption in this mouse strain. Accordingly, ‘SHA 68 + MK-801′-treated mice showed significantly disrupted PPI compared to ‘vehicle + saline’-treated animals (F(1, 68) = 28.15, *p* < 0.0001) at prepulse intensities of 4 dB (*p* < 0.05) and 12 dB (*p* < 0.05).

In C57Bl/6J mice, neither SHA 68 (Student’s *t* test: t(17) = 0.2146, *p* = 0.8326) nor the low dose of MK-801 (0.1 mg/kg) alone changed startle amplitudes when compared to the ‘vehicle + saline’ group (Student’s *t* test: t(18) = 1.379, *p* = 0.1849) (Figure 4C). However, mice treated with both SHA 68 and MK-801 showed significantly higher startle amplitude (Student’s *t* test: *t*(19) = 2.280, *p* = 0.0343). In 129S6/SvEvTac mice, treatment with SHA 68 alone had no effect on startle reflex (Student’s *t* test: *t*(16) = 1.171, *p* = 0.2587; Figure 4D), whereas treatment with MK-801 alone produced a significant increase (Student’s *t* test: *t*(17) = 2.604, *p* = 0.0185). Combined treatment with both SHA 68 and MK-801 also significantly increased startle reflexes when compared with ‘vehicle + saline’-treated mice (Student’s *t* test: *t*(16) = 2.727, *p* = 0.0149).

Together, these data demonstrate a significant role of endogenous NPS signaling for PPI performance and rule out possible confounding effects of genetic manipulations in knockout animals. Contributions of endogenous NPS to PPI may vary between mouse strains, as male 129S6/SvEvTac mice appeared more sensitive to the blockade of NPS signaling than C57Bl/6 males.

### 2.5. Effect of Clozapine in NPSR1 WT and KO Mice

**Figure 5:**
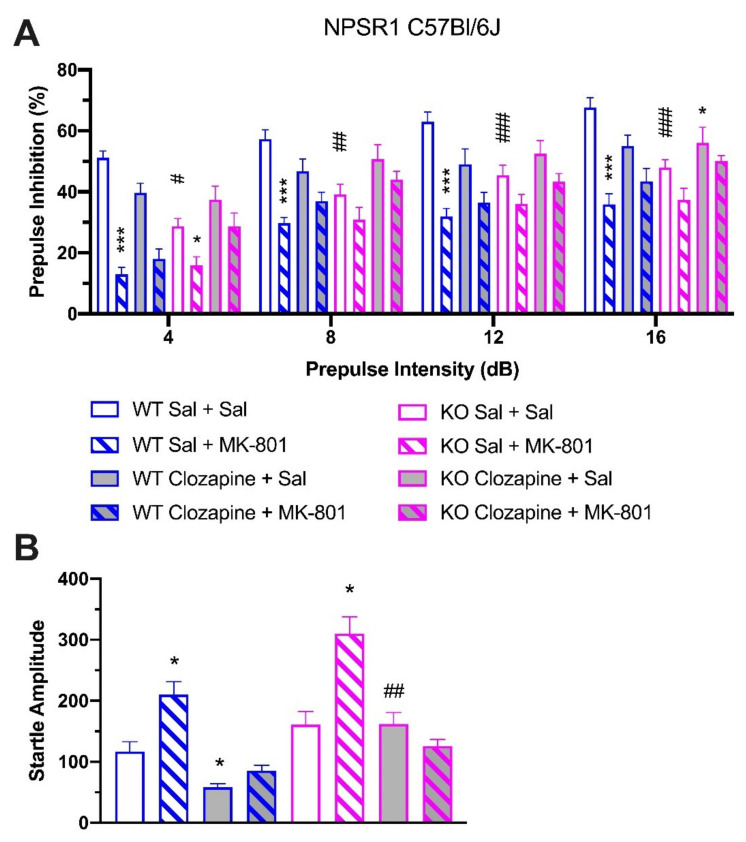
(**A**) Effect of clozapine on PPI disruption in male C57Bl/6J-NPSR1 WT and KO mice. Clozapine significantly attenuated 0.3 mg/kg MK-801-induced PPI impairment in both WT and KO mice and reversed the PPI deficits of NPSR1 KO mice; * *p* < 0.05, *** *p* < 0.01 vs. ‘saline + saline’-treated controls within the same genotype; # *p* < 0.05, ## *p* < 0.01, ### *p* < 0.001 vs. WT ‘saline + saline’-treated controls; Bonferroni’s post hoc test after significant two-way ANOVA. (**B**) Startle reflex after clozapine treatment. MK-801 increased startle amplitude whereas clozapine decreased baseline startle amplitude in WT mice and attenuated startle amplitude in MK-801-treated KO mice. KO mice showed significantly higher baseline startle amplitude than WT mice; * *p* < 0.05 vs. ‘saline + saline’-treated group within the same genotype; ## *p* < 0.01 vs. WT group which received the same treatment; *t*-test. Animal numbers: WT, *n* = 9–12; KO, *n* = 6–7.

Since PPI deficits in schizophrenic patients as well as in pharmacologically-induced animal models can be reversed by treatment with the atypical antipsychotic clozapine, we studied the effect of clozapine (2 mg/kg i.p.) on PPI disruption in male C57Bl/6J-NPSR1 WT and KO mice (Figure 5A). In C57Bl/6J-NPSR1 WT mice, two-way ANOVA indicated robust PPI disruption in animals treated with ‘saline + MK-801′ (0.3 mg/kg, i.p.) compared to ‘saline + saline’-treated mice (F(1, 68) = 131.29, *p* < 0.0001). Two-way ANOVA also showed a significant difference between ‘saline + MK-801′-treated mice and ‘clozapine + MK-801′-treated animals (F(1, 60) = 8.52, *p* = 0.0049), suggesting that clozapine alleviated MK-801-induced PPI impairment in WT mice. In C57Bl/6J-NPSR1 KO mice, treatment with MK-801 alone resulted in a marginally significant reduction in PPI compared to ‘saline + saline’-treated animals (F(1, 48) = 4.03, *p* = 0.0505). Pretreatment with clozapine significantly attenuated MK-801-induced PPI disruption (F(1, 52) = 10.33, *p* = 0.0023). Therefore, clozapine produced significant improvement of PPI in both MK-801-treated WT and KO mice.

Comparing C57Bl/6J-NPSR1 WT and KO groups that had received the same treatment, KO mice showed significantly attenuated PPI compared to WT mice after treatment with saline alone (F(1, 56) = 62.27, *p* < 0.0001), thus replicating the PPI-deficit phenotype as described above. After treatment with MK-801 alone, KO mice displayed lower PPI compared to WT mice, although the difference did not reach significance (F(1, 60) = 3.67, *p* = 0.0602). Interestingly, treatment with clozapine alone produced similar PPI in both WT and KO mice (F(1, 68) = 1.11, *p* = 0.2958), suggesting that clozapine was able to rescue the PPI-deficit phenotype in NPSR1 KO mice.

Startle reflex was also investigated in all genotypes and treatment groups (Figure 5B). Injection of MK-801 increased startle reflex while clozapine attenuated MK-801 effects. In WT mice, treatment with the high dose of MK-801 (0.3 mg/kg) significantly increased the startle reflex (Student’s t test: *t*(17) = 2.473, *p* = 0.0242) whereas treatment with clozapine alone reduced startle amplitudes when compared to saline-treated WT mice (Student’s *t* test: *t*(19) = 2.595, *p* = 0.0178). In KO mice, treatment with MK-801 and clozapine produced significantly lower startle amplitude than in saline-treated animals (Student’s *t* test: *t*(11) = 2.287, *p* = 0.0430). Finally, clozapine-treated KO mice showed significantly higher startle amplitudes than clozapine-treated WT mice (Student’s *t* test: *t*(17) = 3.077, *p* = 0.0068).

### 2.6. Role of NPS Signaling in Attentional Control

**Figure 6:**
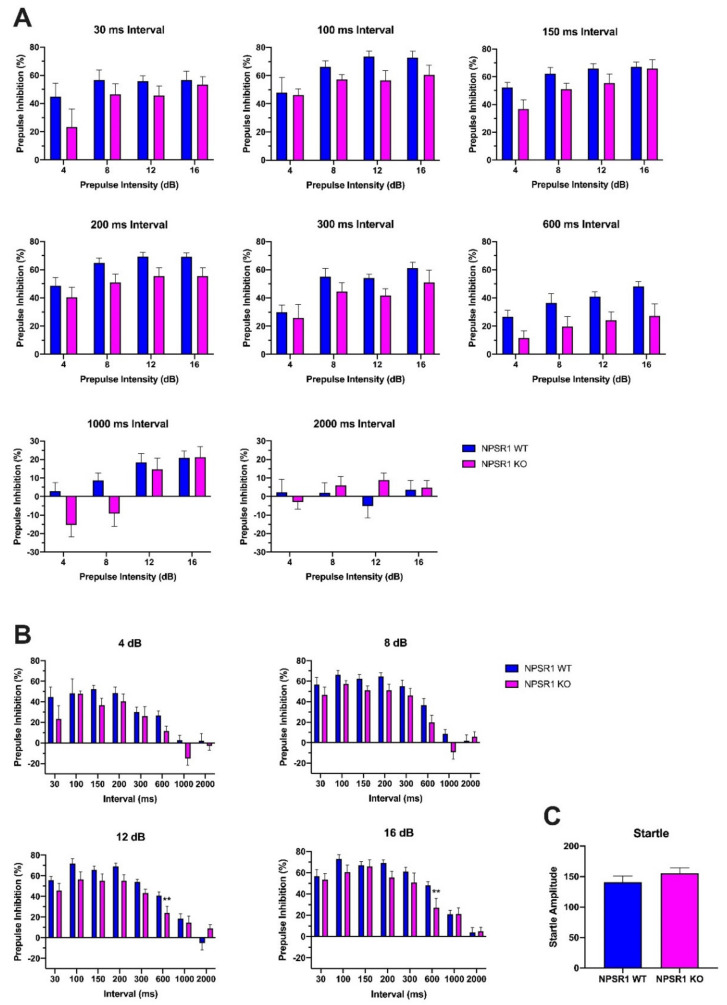
Sustained attention measured in PPI tests with different prepulse-to-pulse interstimulus intervals (ISI). (**A**) PPI of male C57Bl/6J-NPSR1 WT and KO mice at different prepulse-to-pulse intervals. **(B**) PPI of C57Bl/6J-NPSR1 WT and KO mice at different prepulse intensities as a function of increasing ISI. KO mice showed consistently lower PPI compared to WT mice at prepulse-to-pulse intervals between 30 and 1000 ms and increasingly severe PPI deficits with prolonged ISIs. At long ISI (1000 ms) and low prepulse levels KO mice display prepulse facilitation (negative bars); ** *p* < 0.01 vs. WT controls; Bonferroni’s post hoc test after significant two-way ANOVA. (**C**) C57Bl/6J-NPSR1 WT and KO mice showed similar startle amplitude. Animal numbers: WT, *n* = 6–7; KO, *n* = 6–7.

In a next set of experiments, we sought to investigate the role of the NPS system in modulation of attentional control as an integral part of PPI responses. As the startle reflex to the pulse is significantly affected by attention to the prepulse, prolonged prepulse-to-pulse intervals were used to determine whether NPS signaling may be required for sustained attention to the prepulse. Thus, male C57Bl/6J-NPSR1 WT and KO mice were tested in a series of PPI experiments in which 8 different inter-stimulus intervals (ISI) were applied in random order (30, 100, 150, 200, 300, 600, 1000, and 2000 ms) (Figure 6A). Compared to NPSR1 WT mice, NPSR1 KO mice showed increasingly more severe PPI deficits with prolonged ISIs. Two-way ANOVA (prepulse intensity × genotype) revealed significant differences between the genotypes at ISIs of 100 ms (F(1, 44) = 5.29, *p* = 0.0263), 150 ms (F(1, 68) = 8.95, *p* = 0.0039), 200 ms (F(1, 60) = 20.83, *p* < 0.0001), 300 ms (F(1, 72) = 4.13, *p* = 0.0459), and 600 ms (F(1, 48) = 24.82, *p* < 0.0001), with the most dramatic difference at an ISI of 600 ms. The difference between WT and KO mice at 30 ms ISI only approached significance ((F(1, 40) = 4.03, *p* = 0.0515). At 1000 and 2000 ms ISI, two-way ANOVA showed no significant effect of genotype on PPI responses (F_1000_ms_(3, 48) = 0.49, *p* = 0.6905; F_2000_ms_(1, 48) = 2.25, *p* = 0.1402), however, at 1000 ms ISI, KO mice presented prepulse facilitation (PPF) at low prepulse intensities (4 and 8 dB). In other words, the response of NPSR1 KO mice to the startling stimulus was facilitated by the preceding weak non-startling stimulus (4 or 8 dB above background) when ISIs exceeded 600 ms.

Looking at each prepulse level individually, KO mice showed consistently lower PPI than WT mice (Figure 6B). Two-way ANOVA revealed highly significant differences between NPSR1 WT and KO mice at all prepulse levels: 4 dB (F(1, 107) = 7.47, *p* = 0.0073), 8 dB (F(1, 107) = 7.06, *p* = 0.0091), 12 dB (F(1, 107) = 8.57, *p* = 0.0042), and 16 dB (F(1, 97) = 8.84, *p* = 0.0037). Bonferroni post hoc tests indicated significant differences between NPSR1 WT and KO at 12 dB (*p* < 0.01) and 16 dB (*p* < 0.01) when ISI was 600 ms.

As shown in Figure 6C, no difference in startle reflex was detected between C57Bl/6J-NPSR1 WT and KO mice (Student’s *t* test: *t*(38) = 0.3865, *p* = 0.7013).

## 3. Discussion

The present study aimed to address conflicting results about NPS effects on PPI and acoustic startle responses by systematically investigating age, gender, strain, as well as genetic model-dependent and pharmacological influences. Of fundamental importance is our finding of age-dependent PPI improvement in NPSR1 KO mice rather than WT mice, where significant PPI deficits are present only in young adolescent male KO animals (8–12 weeks of age), independent of genetic model or strain (Table 2). We confirmed that female mice, regardless of genetic model, do not display NPS-related PPI differences. PPI deficits in male KO mice can be replicated pharmacologically in WT animals by blocking NPS neurotransmission and can be alleviated by the atypical antipsychotic clozapine. Mechanistically, NPS might positively influence PPI performance by enhancing attentional processes. Therefore, this study provides evidence for age and sex as two major factors of NPS-dependent PPI modulation, which could explain discrepant findings in previous studies.

A major limitation in the study of schizophrenia is the lack of a comprehensive animal model that resembles all aspects of the human disorder. The popularity of PPI as an experimental paradigm for understanding schizophrenia comes from its conceptual linkage to clinical observations that schizophrenia patients are unable to optimally filter or “gate” irrelevant, intrusive sensory stimuli, which results in sensory overload and secondary cognitive fragmentation [20,28,29,30]. As PPI is believed to reflect dysfunctional gating and information processing, it is considered a powerful translational model for both screening antipsychotic compounds [31] and probing the underlying neurobiology and genetics of gating deficits in schizophrenic subjects [32]. While PPI is a reliable, robust quantitative phenotype in human and animal models, investigators are increasingly aware of the many factors affecting the measurement of PPI, such as subject age, strain, and so forth. 

### 3.1. Influences of Age and Strain on PPI

Age-dependent changes in acoustic startle response and PPI have been reported across adolescence and between young adult and aged mice [33,34,35,36]. However, there is scarce evidence on the effect of age on PPI in mice ranging from late adolescence to young adulthood, which corresponds to the peak period of schizophrenia onset in humans. We compared PPI of NPSR1 WT and KO mice at ages between 8 and 20 weeks and found increased PPI in both genotypes when the mice aged, consistent with the observed age effect on PPI in humans [37]. NPSR1 KO mice showed consistently lower PPI than WT animals before week 14, whereas KO and WT groups began to display overlapping PPI performance from that point on. It is possible that young KO mice before week 14 have PPI deficits, as a result of NPS deficiency, while mice of 14 weeks or older have developed sufficient compensatory changes in the neural circuits recruited by PPI. The most significant difference in PPI performance between NPSR1 WT and KO mice occurred around 10 weeks of age and thus all animals in the ensuing experiments were selected to be 8–12 weeks old. Our results seem contrary to those reported in our own previous study [4] and the data reported by Zhu et al. [5], or Fendt et al. [6], in which NPSR1 KO mice showed similar PPI compared to WT animals. Mice in the studies by Duangdao et al. and Fendt et al. were between 8 and 15 weeks and 12 and 25 weeks old, respectively (Table 1). Therefore, it is likely that the wide range of ages in the experimental cohorts masked any difference in PPI between the genotypes that is only visible in young adolescent mice. Indeed, in both studies KO mice displayed consistently lower PPI levels compared to their WT littermates, although never reaching significance. We have demonstrated now that significant PPI deficits are reliably found only in cohorts of younger (<12 weeks of age) mice. It is, however, less clear at present why Zhu et al. [5] did not detect differences in their NPSR1 KO line, since they tested young male mice of a narrow age range (8–10 weeks) that had been sufficiently backcrossed onto a C57Bl/6J background, similar to our strategy. Although Zhu et al. [5] observed consistently lower startle levels in NPSR1 KO mice, they detected equal relative inhibition (% PPI) after using prepulses of 4 and 6 dB over background. Unfortunately, they did not apply a broader range of prepulse intensities that are commonly used in most PPI protocols. In addition, they employed a relatively short ISI of 70 ms between prepulse and pulse presentation, which may have masked a significant genotype difference in PPI performance, since ISI can critically influence PPI as we have observed in this study. In addition to age, strain is also one of the factors affecting PPI in mice [38]. When comparing PPI differences in male NPSR1 WT and KO mice, C57Bl/6J mice exhibited a very significant difference between WT and NPSR1 KO groups (F(1, 104) = 27.28, *p* < 0.0001), whereas 129S6/SvEvTac mice displayed only marginally significant differences between genotypes (F(1, 40) = 3.95, *p* = 0.0537). Such strain-dependent differences in baseline PPI responses have been reported throughout the literature [33,35,38,39].

### 3.2. Gender Differences in PPI

Unlike male mice, however, female mice exhibited similar PPI between NPSR1 WT and KO mice in both C57Bl/6J and 129S6/SvEvTac backgrounds, including after treatment with MK-801. Notably, it has been reported that male patients diagnosed with schizophrenia displayed reduced PPI compared to healthy men, whereas women with schizophrenia showed no deficit in PPI compared to healthy females [40]. However, contrary results have also been reported [41]. NPSR1 KO mice might thus be a translational model to study gender differences in PPI and the underlying neurobiological mechanisms. It should also be noted that male-specific associations of *NPSR1* rs324981 genotypes with panic disorder [8] or stress responsiveness [16] have been reported, suggesting sex-specific functions of the NPS system. Sex differences in PPI have been observed frequently, e.g., in mice [42], rats [43,44,45,46], and human subjects [40,47,48,49,50]. It is proposed that estrogen plays an important role in modulating PPI in female subjects. For instance, PPI levels fluctuate across the menstrual cycle in healthy women, with reduced PPI at peak estrogen levels [48,51], and similar observations have been made in rats [52]. The role of estrogen is further strengthened by the absence of gender effects on PPI in children under 8 years of age [53], in postmenopausal women compared to age-matched men [54], or postmenopausal mice [42]. Therefore, our observation that absence of NPS signaling is associated with PPI deficits exclusively in male mice may suggest an interaction between estrogen and the NPS system. However, female mice in our studies were not synchronized in their estrous cycles and then tested at defined time points.

### 3.3. Sensitivity to Antipsychotic Treatment

Previous studies demonstrated that central administration of NPS enhances dopaminergic neurotransmission in the medial prefrontal cortex [55] and elevated levels of dopamine metabolites in the nucleus accumbens shell were detected after intra-VTA NPS injections [56]. Dense innervation of all major dopaminergic thalamic and hypothalamic cell groups by NPS-immunoreactive fibers was observed in the rat [57] and mouse brain [58]. Furthermore, acute or chronic treatment with both typical and atypical antipsychotic drugs resulted in significant regulation of NPS precursor and NPSR1 transcripts [59,60]. Together, these data suggest important NPS-dopaminergic interactions. Most antipsychotic drugs exert their therapeutic effects via targeting dopaminergic neurotransmission and possess at least moderate dopamine receptor antagonist potency. Therapeutically used antipsychotics are able to reverse PPI deficits in human schizophrenic patients [61].

In order to investigate whether therapeutically successful antipsychotic drugs can alleviate or rescue NPS-dependent PPI deficits, we tested the effect of clozapine in NPSR1 KO mice. Clozapine is an atypical antipsychotic, which has been reported to restore MK-801-induced PPI impairment in rodents [62] and deficient PPI in patients with schizophrenia [63]. We found that clozapine ameliorated MK-801-produced PPI impairment in both NPSR1 WT and KO mice. It is worth noting that clozapine restored PPI in NPSR1 KO mice to levels similar to those of WT mice. This observation is in agreement with previous findings that central administration of NPS mimics the effect of clozapine in MK-801-treated mice [1].

### 3.4. The Role of Attention in PPI Performance

PPI was first proposed to be a pre-attentive and automatic inhibitory mechanism [64]. More recently, ample evidence has shown that PPI can be modulated by attentional processes [23,65,66,67,68,69,70,71]. Given that NPS has been suggested to play a role in cognitive functions and arousal [19,57,72,73,74,75,76,77], we hypothesized that the PPI disruption of NPSR1 KO mice might, at least in part, result from attention deficits caused by the absence of NPS signaling. Therefore, we investigated the effect of a range of ISIs on PPI performance (30–2000 ms ISI). NPSR1 KO mice displayed aggravated PPI disruption with prolonged ISIs at all prepulse intensities. PPI at short ISIs (30 and possibly 60 ms) is believed to reflect largely automatic processing, while PPI at longer ISIs is suggested to reflect both automatic and controlled processing [26,78]. Therefore, PPI reduction at 30 ms ISI suggests deficits of NPSR1 KO mice in automatic processing, whereas increasingly disrupted PPI at longer ISIs (100–600 ms) implies deficits in both automatic and controlled processing, with the deficits of controlled attentional processing further deteriorating PPI disruption caused by deficient automatic processing. WT mice exhibited robustly higher PPI levels compared to NPSR1 KO mice, probably because they are capable of sustained attention to the stimuli, which has been suggested to play an important role in preventing reduced PPI at longer ISIs [79]. Nevertheless, there were no significant differences between WT and NPSR1 KO groups when ISIs were prolonged to 1000 or 2000 ms, which might define the maximal attention span of both genotypes. Collectively, NPS might play a role in the modulation of PPI by influencing attentional control. Given that the mPFC is heavily involved in attentional processes [80,81], the stimulatory effect of NPS on dopamine neurotransmission in the mPFC [55] might be functionally related to its role in attentional control. However, one limitation of the present study on the role of NPS in attentional processes is that we used “passive” PPI methods in which no attentional instructions were given to subjects and attention to the stimuli was unmeasured. Therefore, unconstrained attention across trials and between subjects and groups may complicate the interpretation of findings [25]. Understanding the role of NPS in attentional control thus requires further animal and human studies.

Looking at single prepulse levels across the range of ISIs tested, we found that the shape of PPI stimulus-response curves of NPSR1 WT and KO mice resembled the ISI-dependent PPI differences described for men and women [82], respectively. WT mice constantly display higher PPI than NPSR1 KO mice, and similarly men display higher PPI than women across all ISIs shorter than 1000 ms. At 1000 ms ISI, only NPSR1 KO mice exhibit prepulse facilitation (PPF) at low prepulse intensities (4 and 8 dB). Similarly, at the same ISI, more PPF is observed in women while rather PPI is seen in men [82]. At present, PPF has been less investigated compared to PPI and neuronal mechanisms underlying PPF remain largely unclear. One hypothesis suggests that PPI and PPF might be two aspects of a single underlying process, in which the sensory-motor gate is closed or narrow when ISIs are short (30–600 ms), thus inhibiting processing of newly incoming stimuli (pulse) while the current stimulus (prepulse) is being processed. When ISIs are long enough, however, the gate is open and ready to process the new stimulus because processing of the old stimulus has been completed [82,83,84]. In support of this theory, our results showed a smooth transition from PPI to PPF in NPSR1 KO mice.

### 3.5. Effect on Acoustic Startle Responses

We also investigated whether NPSR1 deficiency could modulate startle reflex in mice of different genetic backgrounds. We found no effect of NPSR1 or NPS precursor genotype on startle reflex, irrespective of gender or strain. In contrast, both Zhu et al. [5] and Fendt et al. [6] reported significantly reduced acoustic startle in C57Bl/6 NPSR1 KO mice. It is thought that the shifts in startle reflex are associated with changes in emotional states, which are known to modulate startle amplitudes [85]. However, causes for the discrepancy observed in our studies and other literature remain unclear. Environmental influences on emotional states (e.g., stress introduced into the animal holding facility, experimental personnel) cannot be excluded, although we did not systematically investigate this possibility. Since we see no differences in startle reflex across different genetic backgrounds, two different genetic models of ablated NPS signaling, or between genders, we believe that NPS may have only a minor influence on neuronal startle circuits, if any.

### 3.6. Conclusions

Collectively, the present results suggest hypofunctional NPS signaling as a contributing factor to PPI deficits. Such a model is also consistent with reports that the low-functioning NPSR1-Asn^107^ variant was significantly associated with schizophrenia in a German cohort, in which decreased verbal memory consolidation was found in homozygous NPSR1-Asn^107^ carriers, who are predicted to possess attenuated NPS neurotransmission [18]. These findings support conclusions of the present study that NPS signaling might play a role in the pathophysiology of schizophrenia and extend our previous findings suggesting a specific effect of NPS on memory consolidation without affecting memory acquisition [19]. The lack of association between NPSR1 genotypes and schizophrenia in a Japanese cohort is currently unexplained, but could be due to insufficient sample size or the large heterogeneity of symptoms used for diagnosis, including the possibility that NPSR1 genotypes might only be associated with particular subforms of the disorder. Obviously, further studies are needed to test this hypothesis.

In conclusion, age-dependent differences in PPI were observed in male mice ranging from late adolescence to young adulthood. Absence of NPS signaling resulted in deficits in PPI or deteriorated MK-801-induced PPI disruption compared to WT controls, exclusively in male mice but independent of genetic background. In addition, blockade of NPSR1 by SHA 68 aggravated MK-801-induced PPI reduction in C57Bl/6J mice and produced PPI deficits on its own in 129S6/SvEvTac mice. Additionally, we found clozapine to improve PPI deficits in NPSR1 KO mice. Moreover, NPSR1 KO mice showed greater PPI deficits when ISIs were prolonged, suggesting NPS might play a role in attentional control that could have a regulatory effect on PPI. Our present study validates the NPS system as an attractive target for the development of novel antipsychotic drugs.

## 4. Materials and Methods

### 4.1. Animals

Male and female mice of both C57Bl/6J and 129S6/SvEvTac background were used for all experiments. NPSR1 KO mice in the 129S6/SvEvTac background were described previously [4,7]. Backcrossing with C57Bl/6J mice for >10 generations yielded congenic mice in a C57Bl/6J background. C57Bl/6J wildtype (WT) mice from commercial sources (Jackson Laboratory, Sacramento, CA, USA; National Cancer Institute, Frederick, MD, USA) were used as controls. A separate line of C57Bl/6J-NPSR1 mice was maintained by heterozygous mating. NPSR1 129S6/SvEvTac mice were maintained by breeding heterozygous offspring as described [4]. NPS precursor KO and WT mice were on a hybrid 129OlaHsd × C57Bl/6J background as described [86] and were maintained by breeding heterozygotes. Breeding schemes followed common guidelines for colony maintenance of transgenic mice to minimize genetic drift. WT littermates were used as controls. For investigation of age effects on PPI performance, one group of mice was tested consecutively in 2-week intervals during 8–20 weeks of age. All other experiments were carried out using naïve mice between 8 and 12 weeks of age for each paradigm or drug.

Mice were group housed under controlled conditions (temperature 21 °C, 12 h light-dark cycle, lights on at 06:00) with water and chow ad libitum. Experiments were carried out between 08:00 and 13:00. All experiments were designed and carried out in accordance with the Guidelines for the Care and Use of Mammals in Neuroscience and Behavioral Research [87], the ARRIVE guidelines for in vivo data reporting [88], and were approved by the Institutional Animal Care and Use Committee (IACUC) of the University of California Irvine (protocol # 2005–2582).

### 4.2. Drugs

MK-801 [dizocilpine maleate; (5R,10S)-(+)-5-Methyl-10,11-dihydro-5H-dibenzo [a,d]cyclohepten-5,10-imine hydrogen maleate] and clozapine [8-Chloro-11-(4- methyl-1-piperazinyl)-5H-dibenzo[b,e] [1,4]-diazepine] were purchased from Sigma (St. Louis, MO, USA) and dissolved in 0.9% saline as vehicle. A stock solution of MK-801 in 0.8% acetic acid was used to prepare working solutions. MK-801 doses were calculated as free base. The NPSR1 antagonist SHA 68 was synthesized as described [89] and dissolved in PBS containing 10% Cremophor EL (Sigma). All drugs were administered by intraperitoneal (i.p.) injection in a total volume of 100 µL/animal.

### 4.3. Drug Administration

NPSR1 WT and KO mice of both C57Bl/6J and 129S6/SvEvTac background received saline or MK-801 (0.1, 0.3 mg/kg) by intraperitoneal (i.p.) injection. Each subject was placed in the PPI test chamber 15 min after saline/MK-801 injection. Both male and female mice were examined for the effect of MK-801 on both genotypes. To investigate NPSR1 antagonist effects, male C57Bl/6J WT mice received either vehicle (PBS, 10% Cremophor EL) or NPSR1 antagonist SHA 68 (50 mg/kg, i.p.), followed by saline or MK-801 (0.1 mg/kg, i.p.) 15 min after the first drug injection. Each subject was placed in the PPI test chamber 15 min after the last injection. To determine the effect of clozapine on MK-801-induced PPI disruption, male C57Bl/6J WT and NPSR1 KO mice were used. Both WT and NPSR1 KO mice received saline or clozapine (2 mg/kg, i.p.) dissolved in 0.9% saline. Systemic injection of saline or MK-801 (0.3 mg/kg, i.p.) followed 15 min after the first drug injection. Each subject was placed in the test chamber 15 min after the second injection.

### 4.4. Prepulse Inhibition

PPI was measured as previously described [1] using automated startle chambers (SR-Lab, San Diego Instruments, San Diego, CA, USA). Briefly, each PPI test session was preceded by a 10 min acclimatization period with 65 dB background noise. Every PPI session included twelve blocks of trials with a total of 70 trials. Blocks 1 and 12 consisted of five pulses alone (a 40 ms 120 dB broadband burst). Blocks 2–11 each contained six trials of each type described below, presented in a pseudo-randomized sequence. Six trial types were presented: a 40 ms broadband 120 dB burst (pulse-alone); four prepulse + pulse trials in which 20 ms-long 69 dB, 73 dB, 77 dB, or 81 dB (4, 8, 12, and 16 dB above background) stimuli (prepulse) preceded the 120 dB pulse by 100 ms (onset to onset); and a no-stimulus trial. Each trial was presented with a false-random intertrial interval (average of 15 s). Startle magnitude was calculated and expressed as the average response to the pulse-alone trials presented during each block of the session. PPI data were calculated and expressed as both percentage and difference scores. Only percentage scores are presented as both measurements yielded comparable results. Percent PPI was derived from the equation: [(pulse alone−(prepulse + pulse))/pulse alone] × 100.

### 4.5. Effect of Interstimulus Interval on PPI

Male C57Bl/6J WT and NPSR1 KO mice were used for PPI tests examining the effect of varying interstimulus intervals (ISI). Blocks one and twelve were the same as described before. Blocks two to eleven each contained six trials of each type described below that were presented in a pseudo-randomized sequence. Six trial types were presented: a 40 ms broadband 120 dB burst (pulse-alone); four prepulse + pulse trials in which the 120 dB pulse was preceded by a 20 ms-long prepulse of 69 dB, 73 dB, 77 dB, or 81 dB (4, 8, 12, and 16 dB above background) at 8 different ISIs: 30, 100, 150, 200, 300, 600, 1000, or 2000 ms after prepulse onset; and a no-stimulus trial. Each trial was presented with a false-random intertrial interval (average of 15 s). Startle magnitude and percent PPI were calculated as described before.

### 4.6. Statistical Analysis

All data are expressed as mean ± standard error of mean (S.E.M.). The normal distribution of sample data was verified by comparing residuals using Anderson–Darling tests, as implemented in GraphPad Prism. Data were examined by analysis of variance (ANOVA) with genotype (one-way) or genotype and time/treatment (two-way) as variables, followed by Bonferroni’s post hoc test, or Dunnett’s post hoc test for pharmacological treatment groups, as appropriate. Unpaired t-tests were used for comparisons of acoustic startle between two genotypes. GraphPad Prism (GraphPad, San Diego, CA, USA) was used for all statistical calculations. Results were considered significant when *p* < 0.05.

## Figures and Tables

**Table 1:**
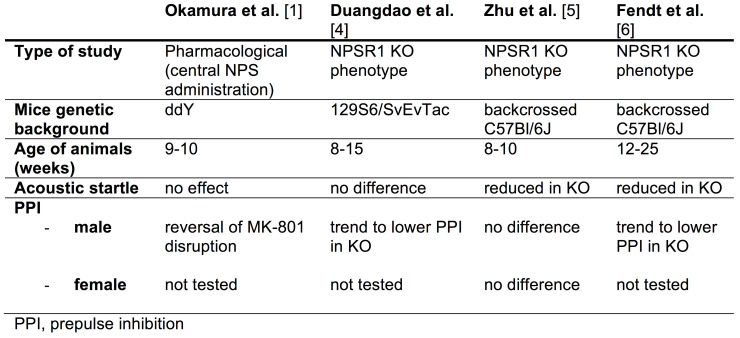
Published NPS/NPSR1 effects on PPI and acoustic startle.

**Table 2:**
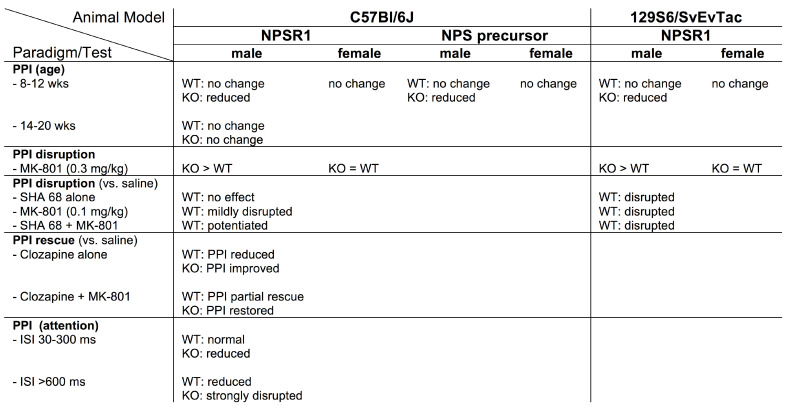
Summary of results.

## Data Availability

The data presented in this study are available in the manuscript.
